# Nitric oxide and superoxide dismutase modulate endothelial progenitor cell function in type 2 diabetes mellitus

**DOI:** 10.1186/1475-2840-8-56

**Published:** 2009-10-30

**Authors:** Saher Hamed, Benjamin Brenner, Anat Aharon, Deeb Daoud, Ariel Roguin

**Affiliations:** 1 Department of Cardiology, Rambam Health Care Campus, Haifa, Israel; 2 Thrombosis & Hemostasis Unit, Rambam Health Care Campus, Haifa, Israel; 3 Department of Endocrinology, Rambam Health Care Campus, Haifa, Israel; 4 The Rappaport Faculty of Medicine, Israel Institute of Technology, Technion, Haifa, Israel

## Abstract

**Background:**

The function of endothelial progenitor cells (EPCs), which are key cells in vascular repair, is impaired in diabetes mellitus. Nitric oxide (NO) and reactive oxygen species can regulate EPC functions. EPCs tolerate oxidative stress by upregulating superoxide dismutase (SOD), the enzyme that neutralizes superoxide anion (O_2_^-^). Therefore, we investigated the roles of NO and SOD in glucose-stressed EPCs.

**Methods:**

The functions of circulating EPCs from patients with type 2 diabetes were compared to those from healthy individuals. Healthy EPCs were glucose-stressed, and then treated with insulin and/or SOD. We assessed O_2_^- ^generation, NO production, SOD activity, and their ability to form colonies.

**Results:**

EPCs from diabetic patients generated more O_2_^-^, had higher NAD(P)H oxidase and SOD activity, but lower NO bioavailability, and expressed higher mRNA and protein levels of p22-phox, and manganese SOD and copper/zinc SOD than those from the healthy individuals. Plasma glucose and HbA1c levels in the diabetic patients were correlated negatively with the NO production from their EPCs. SOD treatment of glucose-stressed EPCs attenuated O_2_^- ^generation, restored NO production, and partially restored their ability to form colonies. Insulin treatment of glucose-stressed EPCs increased NO production, but did not change O_2_^- ^generation and their ability to form colonies. However, their ability to produce NO and to form colonies was fully restored after combined SOD and insulin treatment.

**Conclusion:**

Our data provide evidence that SOD may play an essential role in EPCs, and emphasize the important role of antioxidant therapy in type 2 diabetic patients.

## Background

Hyperglycemia impairs vascular endothelial function, and contributes to the vasculopathies of diabetes mellitus, even with tight glycemic control [1]. Endothelial progenitor cells (EPCs) are circulating cells that originate from the bone marrow, and promote neovascularization at sites of ischemia, hypoxia, injury, or tumor formation [2]. Emerging evidence suggests that there is a negative correlation between the severity of diabetes and EPC count and function [3,4].

The complex pathophysiology of vascular damage in diabetes is not fully understood. EPC dysfunction in type 2 diabetic patients is linked to excessive generation of reactive oxygen species and oxidative stress [5]. Reduced extracellular superoxide dismutase (SOD) activity, also known as copper/zinc SOD (Cu/ZnSOD) is associated with increased vascular oxidative stress, and has been implicated in the endothelial dysfunction of patients with hypertension, congestive heart failure, and coronary artery disease [6]. It has been reported that human EPCs can tolerate oxidative stress because they have high intracellular expression levels of manganese SOD (MnSOD), the enzyme which scavenges superoxide anion (O_2_^-^) [7,8].

Nitric oxide (NO) is a biologically active unstable radical that is synthesized in vascular endothelial cells by NO synthase (eNOS), and its bioavailability depends on the balance between its production and inactivation rates [9]. Decreased NO bioavailability has been proposed as one of the determinants of vascular damage in diabetes. NO can stimulate EPC mobilization from bone marrow stem cell niches to the peripheral circulation so that they can participate in the neovascularization process [10]. Chen and colleagues reported that prolonged exposure of early and late EPCs to high glucose (HG) concentrations reduces their number and proliferative ability, NO bioavailability, and the extent of phosphorylation of eNOS and some members of the PI3-kinase/Akt signaling pathway [11]. Exposure of EPCs to HG concentrations increases NAD(P)H oxidase activity which results in increased O_2_^- ^generation and reduced NO bioavailability because O_2_^- ^inactivates NO and uncouples eNOS [12]. Sorrentino and colleagues demonstrated that NO bioavailability and the *in vivo *reendothelialization capacity of EPCs from diabetic patients can be restored by inactivating NAD(P)H oxidase [13].

In the light of our current knowledge on the causes of EPC dysfunction in type 2 diabetes, we hypothesized that prolonged exposure to hyperglycemia in type 2 diabetes leads to excessive O_2_^- ^generation which, in turn, adversely affects the ability of EPCs to repair the vascular endothelium. This study was undertaken to examine the effects of HG concentrations on EPC function, and the role of SOD in O_2_^- ^inactivation in glucose-stressed EPCs.

## Methods

### Clinical study protocol and subject characteristics

Twenty-three type 2 diabetic patients and 15 healthy age-matched volunteers participated in this study. The patients were selected from the Metabolic Outpatient Clinic of the Rambam Health Care Campus, Haifa, Israel. Clinical data for control participants and patients are presented in Table 1. Diagnosis of type 2 diabetes was the only criterion for inclusion in this study, whereas exclusion criteria included diagnosis of type 1 diabetes, and presence of any of the following self-reported medical conditions: recent surgery, auto-immune diseases, acute or chronic infection and any other unrelated disease. The control participants were selected randomly from healthy volunteers. Medical histories were taken from, and clinical laboratory examinations were performed on all healthy volunteers to confirm that none had clinical or laboratory evidence for diabetes, cardiovascular diseases, inflammatory or auto-immune diseases, obesity or other chronic diseases. The study was approved by the Ethics Committee of the Rambam Health Care Campus, and each participant gave his/her written informed consent.

**Table 1:** Characteristics of the Study Participants

**Characteristic**	**Healthy** **(n = 15)**	**Type 2 Diabetes** **(n = 23)**
Age *(years)*	56.3 ± 2.4	59.6 ± 1.5
Gender *(M/F)*	8/7	17/6
BMI, *(kg/m^2^)*	25.3 ± 1.2	29.7 ± 0.9**
Diabetes duration, *(years)*	__	12.2 ± 0.9
**Clinical history**		
Smoking, *n (%)*	4 *(27)*	7 *(30)*
Hypertension, *n (%)*	__	11 *(48)*
CVD, *n (%)*	__	7 *(30)*
Retinopathy, *n (%)*	__	2 *(9)*
Nephropathy, *n (%)*	__	3 *(13)*
Neuropathy, *n (%)*	__	1 (4)
**Clinical Laboratory Results**		
Plasma glucose levels, *(mg/dl)*	93.4 ± 2.4	221.8 ± 9.5***
HbA1c levels, *(%)*	4.7 ± 0.1	8.6 ± 0.3***
Total cholesterol, *(mg/dl)*	170.4 ± 8.4	178.6 ± 7.2
LDL-cholesterol, *(mg/dl)*	91.6 ± 3.2	107.9 ± 6.5
HDL-cholesterol, *(mg/dl)*	53.2 ± 1.5	51.4 ± 1.7
Triglyceride, *(mg/dl)*	109.5 ± 9.5	127.4 ± 13.2
Creatinine, *(mg/dl)*	0.77 ± 0.03	0.86 ± 0.04
**Medications**		
Insulin, *n (%)*	__	__
Oral antidiabetics, *n (%)*	__	23 *(100)*
-Rosiglitazone, *n (%)*	__	5 *(22)*
-Sulfonylureas, *n (%)*	__	11 *(48)*
-Metformin, *n (%)*	__	13 *(57)*
-Others, *n (%)*	__	4 *(17)*
-Combination, *n (%)*	__	6 *(26)*
ACEIs/ARBs, *n (%)*	__	7 *(30)*
Aspirin, *n (%)*	__	19 *(82)*
Statins, *n (%)*	__	12 *(52)*

Values are presented as mean ± SEM, or number (%) of subjects. Comparisons were made by two-tailed Student's unpaired *t *test or Mann-Whitney test, for non-parametric data. Smokers refer to previous or current smokers. Statistical significance, *P < 0.05, **P < 0.001, ***P < 0.001 versus healthy.

The medical history, results of previous clinical laboratory tests, and medications were obtained from diabetic patient's medical files upon recruitment. Age, gender, BMI, diabetes duration, smoking habit, and the presence of clinical state associated with diabetes complications including hypertension, cardiovascular diseases (CVD), retinopathy, nephropathy, neuropathy, and medication history were recorded (Table 1). All participants underwent a complete metabolic evaluation. Peripheral blood samples (50 mL) were collected from all participants for *ex vivo *EPC assessment, and measurement of plasma glucose and HbA1c levels.

### Isolation, cultivation and characterization of EPCs

**Figure 1:**
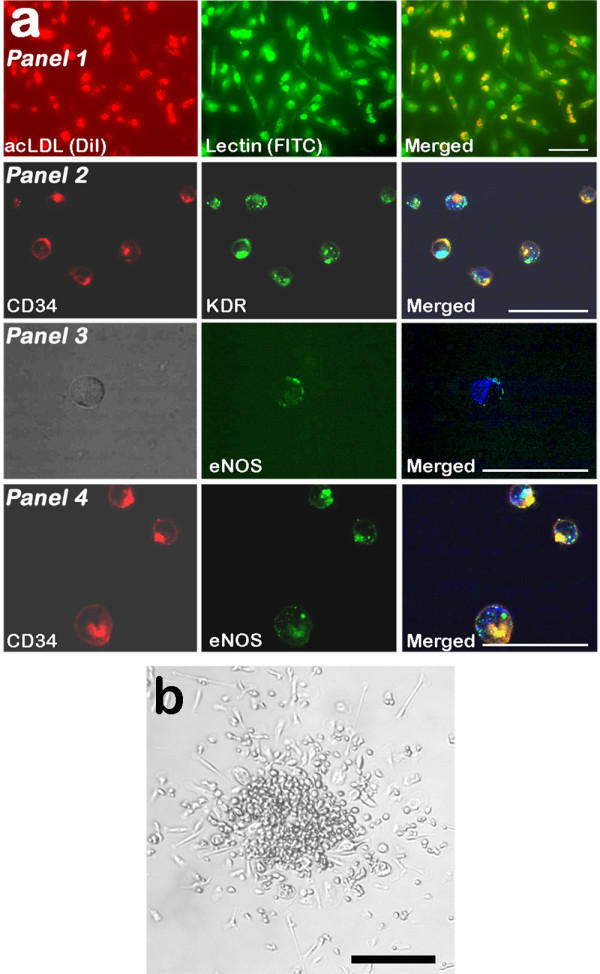
**Endothelial progenitor cell characterization**. Endothelial progenitor cells (EPCs) were cultured for five days. (a) From left to right, *Panel 1*: acetylated LDL uptake by adherent spindle-shaped EPCs, FITC-conjugated lectin UEA-1 binding to the surface of EPCs, and double-positive stained EPCs for acetylated LDL uptake and lectin binding. *Panel 2*: Immunofluorescence detection of the CD34 antigen (red), and KDR (green) on the EPC surface. *Panel 3*: Immunofluorescence detection of eNOS in a single non-stained EPC (green). *Panel 4*: Immunofluorescence detection of the CD34 antigen on the EPC surface (red), and eNOS (green). The EPC nuclei were stained with the blue fluorescent DNA dye DRAQ5™. Scale bare 50 μm. (b) A representative colony of EPCs with a central core of round cells that is surrounded by elongated spindle-shaped cells. Scale bare 100 μm.

EPCs were isolated cultured and characterized, as described previously [14]. Peripheral blood mononuclear cells (MNCs) were isolated by density gradient centrifugation using Lymphoprep™ (Axis-Shield, Oslo, Norway), and then grown in endothelial cell basal medium-2 (EBM-2) (PromoCell GmbH, Heidelberg, Germany) for five days. The EPCs in the cultures were identified as adherent cells that stained double positive for acetylated LDL (acLDL) uptake and the binding of FITC-labeled lectin under a laser scanning confocal microscope. For this purpose, the adherent cells from the EPC cultures were first incubated with 2.2 μg/mL 1,1'-dioctadecyl-3,3,3',3'-tetramethylindocarbocyanine-labeled acLDL (Biomedical Technologies, Inc., MA, USA) for two hours at 37°C. After two hours, the cells were fixed in 2% paraformaldehyde, and then counterstained with 10 μg/mL fluorescein isothiocyanate-labeled lectin from *Ulex europaeus *agglutinin (UEA-1) (Sigma Aldrich, MO, USA). The putative EPCs were stained also for CD34 antigen (CHEMICON Inc., CA, USA), kinase-insert domain receptor (KDR), and eNOS (R&D Systems, MN, USA) (Fig. 1a).

### Determination of EPC count by flow cytometry

Circulating EPCs were analyzed for the expression of the surface antigens CD34 and kinase insert domain receptor (KDR) by two-color direct immunofluorescence flow cytometry [15]. Isolated MNCs were stained with an FITC-conjugated monoclonal antibody against human CD34 (MACS, Bergisch Gladbach, Germany) and a PE-conjugated monoclonal antibody against human KDR (R&D Systems). Identical IgG isotype served as negative controls (R&D Systems). The frequency of double-positive peripheral MNCs was determined by forward and side-scatter fluorescence dot-plot analysis of a 5 × 10^5 ^cell sample using a FACS Calibur analyzer (Becton Dickinson, NJ, USA). Data were processed using the Macintosh CELLQuest software program (Becton Dickinson).

### Experimental protocols

Isolated EPCs from healthy volunteers were maintained in EBM-2 with either 5.5 mmol/L D-glucose (NG) or 25 mmol/L D-glucose (HG) for five days before they were used in the following two experimental protocols. High L-glucose (25 mmol/L) was used as an osmolarity control.

#### Protocol 1

In order to establish whether NO production and O_2_^- ^generation were impaired in glucose-stressed EPCs, their NO production and O_2_^- ^generation were compared to that measured in non-stressed EPCs in the presence and absence of 100 μM/mL L-NAME (a non-specific NOS inhibitor) and 10 μM/mL apocynin (an NAD(P)H oxidase inhibitor).

#### Protocol 2

In order to establish whether the impaired NO production of glucose-stressed EPCs could be restored by increasing EPC glucose utilization or by exogenous SOD, the effects of insulin, which is known to increase eNOS expression in endothelial cells [15], and SOD supplementation on NO production, O_2_^- ^generation, and their capacity to form colonies were determined by treating glucose-stressed EPC with 100 μU/mL insulin for five days, 250 U/mL SOD for two days, or 250 U/mL SOD and 100 μU/mL insulin for two and five days.

### EPC colony-forming unit counts

The ability of EPCs to form colonies was used as a marker of proliferation [16]. An EPC colony-forming unit (CFU) comprises a central core of round cells that is surrounded by elongated spindle-shaped cells (Fig. 1b). The numbers of colonies were counted manually after five days of EPC culture, and expressed as the average number of CFUs per well.

### Measurement of NAD(P)H oxidase activity and O_2_^- ^generation

The generation of O_2_^- ^from EPCs was measured using a lucigenin-enhanced chemiluminescence assay [17]. Cultured EPCs (1 × 10^6 ^cells/ml) after the various treatments were first lysed, and then 100 μM NAD(P)H in order to generate O_2_^-^, followed by 5 μM lucigenin were added to each lysate. The amount of generated O_2_^- ^in each sample was quantified by measuring the intensity in a fluorescence spectrophotometer, and was expressed as a percentage of that in the lysates of healthy or non-stressed EPCs.

NAD(P)H oxidase activity was measured in similar experiments. Chemiluminescence was recorded every 15 s for 10 min. The lucigenin chemiluminescence was expressed as counts per min per 10^6 ^cells. NAD(P)H (final concentration 100 μmol/l) was added after measurement of background lucigenin chemiluminescence and measurement were performed for another 10 min. the difference between the values obtained before and after adding NAD(P)H was calculated and it represented the activity of NAD(P)H oxidase, and was expressed as a percentage of that in the lysates of healthy EPCs.

### Measurement of SOD activity

The Superoxide Dismutase (SOD) Detection Kit™ (Cell Technology Inc., CA, USA) was used to determine SOD activity. After detaching and lysing the adherent EPCs, aliquots (150 μl) of cell lysate were transferred to each well of a 96-well microplate that contained tetrazolium salt (WST-1) and xanthine oxidase, and then incubated at 37°C for 20 minutes. The rate of WST-1 formazan formation (inversely proportional to SOD activity) was then measured in a microplate reader at OD_440_.

### Measurement of NO production

NO production by cultured EPCs can be determined from the NO content in the culture medium [18]. EPCs were treated with either 5 μM bradykinin (Sigma) or 100 μM L-arginine (Sigma) for 30 minutes at 37°C, and then 1 μM/mL 4, 5-diaminofluorescein (DAF-2) (Cell Technology Inc.) was added to the EPC cultures. NO content in the EPC culture medium was then measured in a fluorescent spectrophotometer at an excitation wavelength of 488 nm and an emission wavelength of 515 nm. The NO content in medium of the glucose-stressed EPCs was expressed as the percentage of the NO content in the medium of the unstressed EPCs.

### Analysis of mRNA and protein expression

Total RNA was extracted from EPCs using the MasterPure RNA purification kit (EPICENTER Biotechnologies, Madison, WI, USA). For each sample, approximately 50 ng of RNA were reversed transcribed in triplicate using Absolute QPCR Mixes Reverse Transcription Reagents and the Verso cDNA Reverse Transcriptase kit, both of which were purchased from ABgene, UK. Real-time PCR was conducted to examine the levels of human p22-phox; a membrane-bound component of NAD(P)H oxidase, MnSOD and Cu/ZnSOD in healthy and diabetic EPCs. Quantitative amplification of the p22-phox, Cu/ZnSOD and MnSOD cDNA was performed using SYBR Green I (Molecular Probes, Eugene, OR) for 35 cycles that consisted of heat denaturation, annealing and extension using Rotor-Gene 6000 (Corbett Life Science, Sydney, Australia). Levels of human p22-phox, Cu/ZnSOD and MnSOD mRNA were normalized against GAPDH mRNA and expressed as the average of mean percentage of those from healthy EPCs.

Protein extracts for western blot analysis were prepared by lysing EPCs from healthy and diabetic patients in RIPA Lysis Buffer (Millipore, MA, USA) that contained a protease inhibitor. The lysates were resolved on SDS-PAGE gels, and then transferred to polyvinyldene difluoride membranes by electroblotting. The membranes were first incubated with monoclonal antibodies against p22-phox, Cu/ZnSOD and MnSOD (Santa Cruz, CA, USA), and then with an appropriate horseradish peroxidase-conjugated secondary antibody. An antibody against β-actin (Santa Cruz) was used to normalize protein loading. The resultant bands were quantified by densitometry. The results were expressed as the average of mean percentage of lysates from healthy EPCs.

### Statistical analysis

Data are expressed as mean value or percentage ± standard error of the mean (SEM). The unpaired Student's *t *test was used to compare the data from two groups, and one-way analysis of variance was used when there were more than two groups. Pearson's correlation coefficient was used to determine the relationships between NO production and O_2_^- ^generation by the patient's EPCs, and his/her individual plasma glucose and HbA1c levels, and between SOD activity, and O_2_^- ^generation and NO production. Relationships between risk factors or medications and NO production from EPCs were examined by multivariate analysis. The level of statistical significance was set at 5%. A computerized statistical software program (Prism version 5.0, GraphPad, CA, USA) was used to analyze the data.

## Results

### Effect of diabetes on EPC

**Figure 2:**
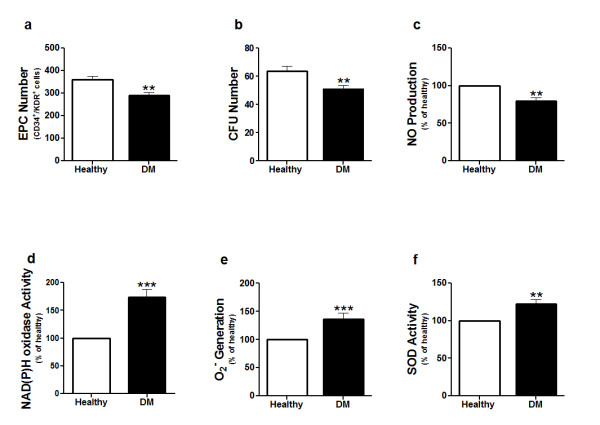
**Endothelial progenitor cell number and function**. Endothelial progenitor cells (EPCs) from diabetic patients and healthy individuals were cultured for five days. (a) Circulating EPCs were labeled with CD34 and KDR cell surface antigens, and then identified by flow cytometry. The bars represent the number of circulating EPCs in the two study groups. (b) The numbers of colony-forming units (CFUs) of EPCs were counted manually in the two study groups. (c) Nitric oxide (NO) content in the medium was determined by measuring the intensity of DAF-2 fluorescence in the EPC culture medium. (d) NAD(P)H oxidase activity in EPCs from type 2 diabetes patients and healthy individuals and (e) Superoxide anion (O_2_^-^) generation by EPCs from type 2 diabetic patients and healthy individuals were measured by the lucigenin-enhanced chemiluminescence assay. (f) SOD activity in EPCs of type 2 diabetic patients and healthy individuals. The results in c, d, e, and f are expressed as a percentage of fluorescence intensity of the healthy group. Data are expressed as mean or percentage ± SEM. *P < 0.05, **P < 0.01, ***P < 0.001. DM represents diabetic patients.

Plasma glucose and HbA1c levels, and BMI in the diabetic patients were significantly higher than those in the healthy volunteers (Table 1). The circulating EPC count of diabetic patients and the proliferative ability of the EPCs were significantly lower than those of the EPCs from healthy volunteers (Fig. 2a and Fig. 2b). EPCs of the diabetic patients produced less NO in response to bradykinin stimulation than that produced by EPCs from healthy volunteers (Fig. 2c). NAD(P)H oxidase activity (Fig. 2d), O_2_^- ^generation (Fig. 2e) and SOD activity (Fig. 2f) in EPCs from the diabetic patients were higher than those in EPCs from healthy volunteers.

**Figure 3:**
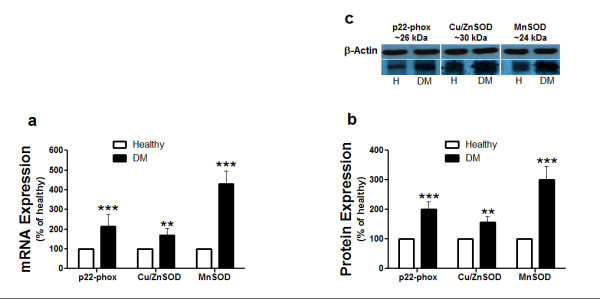
**mRNA and protein expression of SODs and p22-phox in EPCs**. Total RNA and protein of EPCs from type 2 diabetic patients and healthy volunteers were isolated and the mRNA and the protein expressions of a membrane-bound component of NAD(P)H oxidase; p22-phox and the antioxidant enzymes; Cu/ZnSOD, and MnSOD were assessed. (a) Comparison of mRNA expression between p22-phox, Cu/ZnSOD, and MnSOD in EPCs of healthy volunteers (white bars) and type 2 diabetic patients (black bars). (b) Comparison of protein expression between p22-phox, Cu/ZnSOD, and MnSOD in EPCs of healthy volunteers (white bars) and type 2 diabetic patients (black bars). (c) Representative bolts. Blots were scanned and expression of p22-phox, Cu/ZnSOD, and MnSOD was quantified by densitometric analysis and normalized with β-actin. Data are expressed as mean or percentage ± SEM. *P < 0.05, **P < 0.01, ***P < 0.001. DM represents diabetic patients.

EPCs from diabetic patients express significantly higher levels of p22-phox, and of the antioxidative enzymes Cu/ZnSOD and MnSOD compared to EPCs from healthy volunteers (Fig. 3a). The expression of p22-phox, Cu/ZnSOD and MnSOD was further elucidated by western blot analysis. As shown in fig. 3b, the protein expression of p22-phox, Cu/ZnSOD and MnSOD was significantly higher in EPCs from diabetic patients compared with EPCs from healthy volunteers (Fig. 3b).

### Relationship between NO bioavailability in EPCs and diabetes

**Figure 4:**
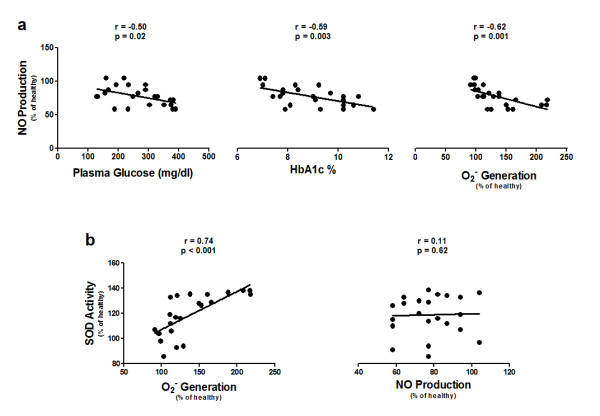
**Relationship between NO production and O_2_^-^generation by EPCs, and the plasma glucose and HbA1c levels in type 2 diabetic patients**. (a) Scatter plot of the relationship between NO production and O_2_^- ^generation by EPCs from type 2 diabetic patients and the individual patient's plasma glucose and HbA1c levels at the time of blood collection. (b) Scatter plot of the relationship between SOD activity in EPCs from type 2 diabetic patients and O_2_^- ^generation, and NO production of their EPCs.

The plasma glucose and HbA1c levels in the diabetic patients, and O_2_^- ^generation by their EPCs were correlated negatively with NO production by their EPCs (Fig. 4a). In contrast, SOD activity of the EPCs of diabetic patients was correlated positively with O_2_^- ^generation by their EPCs, but not with their NO production (Fig. 4b).

**Figure 5:**
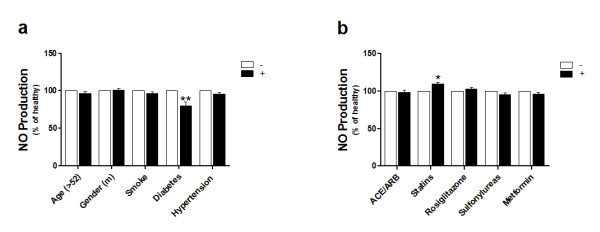
**Effect of individual risk factors and medications on NO production from EPCs**. (a) Effect of individual risk factors on NO production by EPCs of all diabetic patients. (b) Association between the individual medications that was taken by the type 2 diabetic patients and NO production by their EPCs. Increased NO production by EPCs was associated with the use of statins only. Data are expressed as mean percentage ± SEM. *P < 0.05, **P < 0.01.

Of all risk factors that may affect NO production by EPCs, diabetes was the only one that was significantly associated with reduced NO production (Fig. 5a). Furthermore, we found that increased NO production by EPCs was associated only with the use of statins by the diabetic patients (Fig. 5b).

### High glucose effects on EPCs

**Figure 6:**
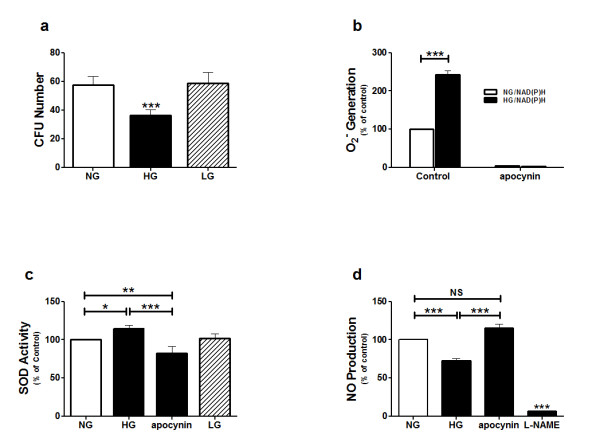
**Assessment of glucose-stressed EPC cultures**. (a) Number of colonies (CFUs) of non-stressed EPCs (normal glucose (5 mmol/L) (NG)), glucose-stressed EPCs (25 mmol/L D-glucose (HG)), and an osmolarity control (25 mmol/L L-glucose (LG)). (b) Superoxide anion (O_2_^-^) generation in non-stressed and glucose-stressed EPCs in the absence or presence of apocynin. (c) SOD activity in non-stressed EPCs (NG), glucose-stressed EPCs (HG), glucose-stressed EPCs treated with apocynin, and an osmolarity control (25 mmol/L L-glucose (LG)). (d) NO levels in non-stressed EPCs (NG), glucose-stressed EPCs (HG), glucose-stressed EPCs after being treated with apocynin, an inhibitor of NAD(P)H oxidase, and glucose-stressed EPCs after being treated with L-NAME, a non-specific inhibitor of NOS. Data are expressed as mean percentage ± SEM or mean number ± SEM of triplicate measurements in each sample from four independent tests. *P < 0.05, **P < 0.01, ***P < 0.001 vs. non-stressed EPCs (control). NS represents non-significant.

The proliferative capacity of EPCs was significantly impaired in glucose-stressed EPCs when compared to that of non-stressed EPCs (Fig. 6a). This impairment was not due to an increase in osmolarity of the medium because the proliferative capacity of non-stressed EPCs and those exposed to L-glucose was the same.

Glucose-stressed EPCs generated significantly higher O_2_^- ^levels than that in non-stressed EPCs. The increased O_2_^- ^generation could be completely abrogated by apocynin (Fig. 6b). SOD activity in glucose-stressed EPCs was significantly higher than that in non-stressed EPCs or EPCs that were exposed to high L-glucose concentration. Treatment of the glucose-stressed EPCs by apocynin decreased significantly SOD activity (Fig. 6c).

NO production by glucose-stressed EPCs was reduced significantly when compared to that of non-stressed EPCs. Apocynin treatment restored NO production, whereas L-NAME treatment abrogated NO production (Fig. 6d).

### The effect of insulin and SOD on glucose-stressed EPCs

**Figure 7:**
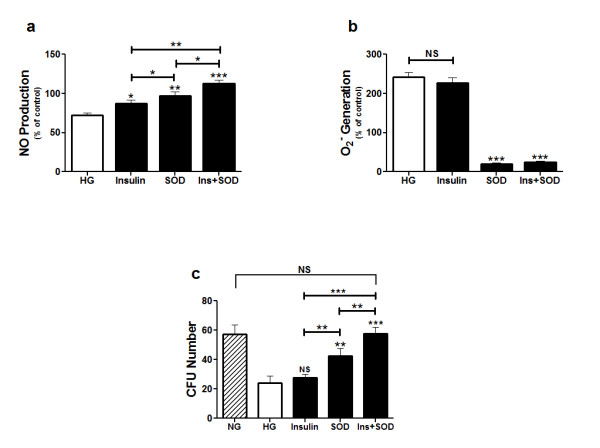
**Insulin and SOD effects on glucose-stressed EPC cultures**. Glucose-stressed EPCs were treated with insulin, SOD, or both. (a) Effect of insulin, SOD, or both on NO production. (b) Effect of insulin, SOD, or both on O_2_^- ^generation. (c) Effect of insulin, SOD, or both on the number of EPC colonies. Data are expressed as mean percentage ± SEM or mean number ± SEM of triplicate measurements in each sample from four independent tests. *P < 0.05, **P < 0.001, ***P < 0.001 vs. glucose-stressed EPCs. NS represents non-significant.

The impaired NO production of glucose-stressed EPCs was restored by adding either SOD, insulin, or both to the EPC cultures (Fig. 7a). Treating glucose-stressed EPCs with insulin had no effect on O_2_^- ^generation, whereas treating them with either SOD or SOD and insulin completely abolished O_2_^- ^generation (Fig. 7b). The impaired proliferative ability of glucose-stressed EPCs was restored partly by treating them with SOD, but not with insulin. Complete restoration of this ability occurred when the glucose-stressed EPCs were treated with insulin and SOD concomitantly (Fig. 7c).

## Discussion

The main finding of this investigation is the important role of SOD in modulating EPC function under hyperglycemic conditions. This conclusion is supported by our finding that the addition of SOD restores NO production and the proliferative ability of glucose-stressed EPCs. This restorative action of SOD may be due to SOD scavenging O_2_^-^, thereby increasing NO bioavailability and/or preventing the uncoupling of eNOS.

Reduced EPC counts and function are associated with poor cardiovascular outcomes [16,19]. In uncontrolled diabetes, reduced EPC count and functionality is associated with hyperglycemia [3,4]. Moreover, eNOS activity and NO bioavailability in EPCs is reduced following prolonged exposure to hyperglycemia [11]. Ozuyaman and colleagues demonstrated that EPC mobilization and function require NO [10]. In addition, Landmesser et al [20] demonstrated that the improvement of EPC survival and mobilization by statins requires eNOS in order to induce myocardial neovascularization in mice. Therefore, it seems that the NO which is produced by EPCs themselves creates a favorite and optimal environment to promote their mobilization and expansion.

Endothelial dysfunction is characterized by low bioavailability of endothelium-derived NO which is itself an independent predictor of future cardiovascular events. The extent of the interaction between NO and O_2_^- ^is thought to be important in the development of endothelial dysfunction because the resultant product, peroxynitrite, can inactivate soluble guanylyl cyclase [21]. In addition, increased generation and inadequate removal of O_2_^- ^can result in oxidative stress, and the development of endothelial dysfunction. The results of recent studies suggest that reduced extracellular SOD activity is closely associated with increased vascular oxidative stress, and has been implicated in the endothelial dysfunction of patients with hypertension [22], congestive heart failure, and coronary artery disease [6]. Human EPCs have high intracellular expression levels of MnSOD, and EPCs are dependent on this level of expression to protect themselves against oxidative stress [7,8]. Tao and colleagues demonstrated that augmenting Cu/ZnSOD expression in human EPCs by shear stress can accelerate O_2_^- ^neutralization. Indeed, they suggested that this O_2_^- ^neutralization leads to increased local NO bioavailability, thereby enhancing the EPC repair potential in the vascular system [23].

We demonstrated that O_2_^- ^generation in glucose-stressed EPCs is higher than that of non-stressed EPCs, and some of this O_2_^-^inactivated NO because the rate of NO production increased after inhibiting NAD(P)H oxidase activity with apocynin. We showed also that NAD(P)H oxidase activity, O_2_^- ^generation and SOD activity are increased by EPCs from the diabetic patients. Indeed, we found that SOD activity of EPCs from diabetic patients was positively correlated with their level of O_2_^- ^generation but not with their level of NO production. The increased SOD activity which accompanied the increased O_2_^- ^generation in these EPCs may account for the intrinsic ability of EPCs to withstand the oxidative stress produced by O_2_^-^. We confirmed this correlation by demonstrating (a) an increase in mRNA and protein expression of p22-phox and both Cu/ZnSOD and MnSOD in EPCs from diabetic patients compared with EPCs from healthy volunteers, and (b) a decrease in SOD activity in glucose-stressed EPCs that were treated with apocynin. In fact, inhibition of O_2_^- ^generation by inhibiting NAD(P)H oxidase activity with apocynin confirms that high O_2_^- ^causes for increased SOD activity in glucose-stressed EPCs. The EPCs of diabetic patients remained however with low NO production and high O_2_^- ^levels despite high SOD activity. The increased SOD activity of the EPCs of diabetic patients may be not sufficient to neutralize the high O_2_^-^levels caused by diabetes. We showed that there is a negative correlation between plasma glucose levels and HbA1c levels and the levels of NO production by EPCs from patients with diabetes. When plasma glucose levels and HbA1c levels increase, less NO will be produced by the EPCs of the diabetic patients, and this may account for their increased risk of developing cardiovascular disease. In addition, we found that the level of O_2_^- ^generation by EPCs of diabetic patients was inversely correlated with their level of NO production. Increased generation of O_2_^- ^results in an augmented interaction between O_2_^- ^and NO, which in turn leads to accelerated inactivation of NO and its reduced bioavailability in EPCs.

Glucose stress in EPCs could generate O_2_^- ^via several processes that include glucose auto-oxidation, increased protein kinase C and NAD(P)H oxidase activity [12]. For example, inhibiting NAD(P)H oxidase activity in EPCs from diabetic patients can restore their NO bioavailability and function [13]. Accumulating data have shown that statin therapy can inhibit NAD(P)H oxidase activation and increase NO bioavailability in diabetes [24-26]. We found that increased NO production by EPCs was associated only with the use of statins by the diabetic patients. The increased O_2_^- ^generation by EPCs could be due to either increased production of O_2_^-^, exhaustion of the enzymatic antioxidant systems, or both. Ohshima and colleagues have demonstrated that antioxidant therapy with SOD in diabetic mice reduced oxidative stress, and increased their EPC count and potential to differentiate into endothelial cells [27]. In our study, we showed that treating glucose-stressed EPCs with SOD restored their NO production and proliferative ability, and this result suggests a protective role for SOD. However, it is possible that adding SOD changed the balance between NO and O_2_^-^. Less NO was inactivated by O_2_^-^, and the overall result was an increase in NO bioavailability of EPCs.

In our study, we stimulated glucose utilization by EPCs using insulin in order to highlight the role of SOD on NO production by EPCs. Insulin enhances eNOS mRNA and protein expression in endothelial cells without affecting oxidative stress [28]. Therefore, we used insulin rather than other antidiabetic drugs such as rosiglitazone, because these drugs are reported to decrease NAD(P)H oxidase activity and oxidative stress in endothelial cells [29]. However, we found that insulin treatment did not change O_2_^- ^levels in glucose-stressed EPCs but restored partially NO production. Although NO production in glucose-stressed EPCs was enhanced by insulin, their proliferative ability remained impaired. We propose that insulin could not restore the proliferative ability of glucose-stressed EPCs because of increased O_2_^- ^levels that was not neutralized and which in turn decreases NO bioavailability.

Although the exposure time of EPCs to HG concentrations in our *in vitro *assays is much shorter than that of EPCs to hyperglycemia in chronic diabetic patients, our finding that treating glucose-stressed human EPCs with SOD restored their functionality is in agreement with that of Ohshima and colleagues in diabetic mice. Alterations in either O_2_^- ^generation and/or SOD activity/expression can markedly alter NO bioavailability in EPCs. Therefore, SOD supplementation could be an excellent strategy to reduce excessive O_2_^- ^production by EPCs and restore their repair potential. Future studies whose aim is to explore the effect of exogenous SOD supplementation on the preservation of endothelial function in diabetes are needed in order to confirm this suggestion.

## Conclusion

The results of this study suggest that increased oxidative stress plays an important role in EPC dysfunction in diabetes. We found evidence for separate, but complementary, effects of SOD and insulin treatment on the functions of glucose-stressed EPCs. These findings emphasize the important role of antioxidant therapy in diabetic patients.

## Abbreviations

CFU: colony forming unit; DAF: 4, 5-diaminofluorescein; EBM: endothelial-cell basal medium; EPC: endothelial progenitor cell; HG: high glucose; KDR: kinase insert domain receptor; L-NAME: L-nitro amino-methyl ester; MNC: mononuclear cell; NO: nitric oxide; NOS: nitric oxide synthase; O_2_^-^: superoxide anion; SOD: superoxide dismutase.

## Competing interests

The authors declare that they have no competing interests.

## Authors' contributions

Each author contributed significantly to the submitted work, and have read and approved the manuscript before its submission. The contribution(s) of each author are as follows: SH contributed to the conception, design, statistical analysis and interpretation of data, and conceived of and wrote the article as well as drafting and final approval of the manuscript submitted. BB, AA, DD and AR contributed to the analysis, interpretation of data, as well as to the revision critically for important intellectual content and final approval of the manuscript submitted.
